# Who Is in There? Exploration of Endophytic Bacteria within the Siphonous Green Seaweed *Bryopsis* (Bryopsidales, Chlorophyta)

**DOI:** 10.1371/journal.pone.0026458

**Published:** 2011-10-18

**Authors:** Joke Hollants, Olivier Leroux, Frederik Leliaert, Helen Decleyre, Olivier De Clerck, Anne Willems

**Affiliations:** 1 Laboratory of Microbiology, Department of Biochemistry and Microbiology, Ghent University, Ghent, Belgium; 2 Phycology Research Group, Department of Biology, Ghent University, Ghent, Belgium; 3 Pteridology Research Group, Department of Biology, Ghent University, Ghent, Belgium; East Carolina University, United States of America

## Abstract

Associations between marine seaweeds and bacteria are widespread, with endobiotic bacterial-algal interactions being described for over 40 years. Also within the siphonous marine green alga *Bryopsis*, intracellular bacteria have been visualized by electron microscopy in the early ‘70s, but were up to now never molecularly analyzed. To study this partnership, we examined the presence and phylogenetic diversity of microbial communities within the cytoplasm of two *Bryopsis* species by combining fluorescence *in situ* hybridization (FISH), denaturing gradient gel electrophoresis (DGGE) and 16S rRNA gene clone libraries. Sequencing results revealed the presence of *Arcobacter*, Bacteroidetes, Flavobacteriaceae, *Mycoplasma*, *Labrenzia*, Phyllobacteriaceae and Xanthomonadaceae species. Although the total diversity of the endobiotic communities was unique to each *Bryopsis* culture, Bacteroidetes, *Mycoplasma*, Phyllobacteriaceae, and in particular Flavobacteriaceae bacteria, were detected in several *Bryopsis* samples collected hundreds of kilometres apart. This suggests that *Bryopsis* closely associates with well-defined endophytic bacterial communities of which some members possibly maintain an endosymbiotic relationship with the algal host.

## Introduction

Marine macroalgal-bacterial associations range from beneficial, harmful or neutral, over obligate or facultative, to ecto- or endophytic interactions [1]. Elaborating the latter, endobiotic associations between marine macroalgal hosts and bacteria have been reported over the past 40 years. Besides reports of bacterial endosymbionts associated with red algal galls [2]–[4], endophytic bacteria have been microscopically observed in the vacuolar as well as cytoplasmatic regions of various bryopsidalean green algae, including *Bryopsis*, *Penicillus*, *Halimeda*, *Udotea* and *Caulerpa* [5]–[10]. These seaweeds are composed of a single, giant tubular cell and form an interesting biotic environment for bacterial communities. The giant cell contains millions of nuclei and chloroplasts in a thin cytoplasmic layer surrounding a large central vacuole. The cytoplasm typically exhibits vigorous streaming, enabling transport of nutrients, organelles and various biomolecules across the plant [11]. In *Bryopsis* ‘bacteria-like particles’ have been visualized in the cytoplasm by means of transmission electron microscopy in vegetative thalli as well as in the gametes, the latter suggesting vertical transmission of the endophytic bacteria [5]. This implies a stable and specific relationship between the algal host and its endobionts in which both partners may provide mutualistic ecological benefits. To date, the diversity of the intracellular microbial communities associated with *Bryopsis* remains unidentified. Up till now investigations of the bacterial endophytic diversity of siphonous macroalgae have been limited to *Caulerpa* species and revealed endosymbiotic Alphaproteobacteria with the potential to photosynthesize, detoxify and/or fix nitrogen [9], [10]. The endophytic bacteria in *Bryopsis* may similarly possess ecologically significant functions and bioactive potential since *Bryopsis* is a substantial source of bioactive compounds such as therapeutic kahalalides which may be of bacterial origin [12], [13].

In order to explore these algal-endophytic bacterial interactions, we previously developed a surface sterilization protocol for the complete elimination of bacterial epiphytes from the *Bryopsis* surface [14]. We showed that *Bryopsis* samples treated with a combined chemical and enzymatic approach (i.e. a mixture of cetyltrimethylammonium bromide (CTAB) lysis buffer, proteinase K and the bactericidal cleanser Umonium Master) remained intact after sterilization and showed no remaining bacterial fluorescence on their surface when stained with a DNA fluorochrome. Successful 16S rRNA gene DGGE analysis following this surface sterilization treatment showed that endophytic DNA was still present within the sterilized *Bryopsis* samples, allowing specific molecular processing of the endophytes [14].

In this study, we verified the presence of bacteria inside two *Bryopsis* species from the Mexican west coast by a combination of fluorescence *in situ* hybridization (FISH), denaturing gradient gel electrophoresis (DGGE) and clone libraries.

## Materials and Methods

### Ethics Statement

No specific permits were required for the described field studies, i.e. the collection of algal samples from the Mexican west coast, because marine algae are not included in the Convention on International Trade in Endangered Species of Wild Fauna and Flora (CITES, http://www.cites.org/eng/disc/species.shtml). The authors confirm that the location is not privately-owned or protected in any way and that the field studies did not involve endangered or protected species.

### Algal material

**Figure 1:**
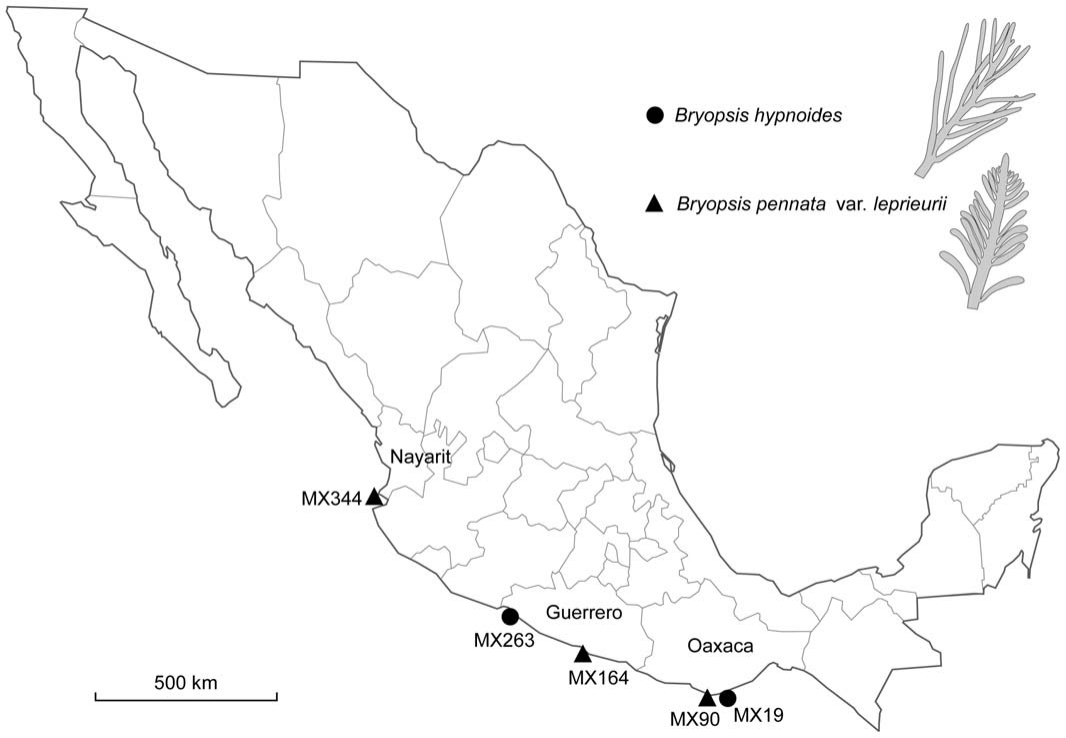
*Bryopsis* sampling sites along the Pacific Mexican coast. *Bryopsis hypnoides* (•) and *Bryopsis pennata* var. *leprieurii* (▴) samples were collected from following sites: Playa el Pantheon (MX19), Mazunte Beach (MX90), Acapulco (MX164), Playa las Gatas (MX263) and Playa Careyero (MX344).

Five *Bryopsis* specimens were collected in February 2009 along the Pacific Mexican coast at different sites located between Mazunte Beach (Oaxaca, southwest Mexico) and Playa Careyero (Nayarit, central Mexico) (Figure 1). These five samples were classified in two different species with samples MX19 and MX263 representing *Bryopsis hypnoides* J.V. Lamouroux and MX90, MX164, and MX344 representing *Bryopsis pennata* J.V. Lamouroux var. *leprieurii* (Kützing) Collins and Hervey individuals. After sampling, living specimens were rinsed with sterile seawater and transferred to the laboratory in plastic vessels containing a small amount of sterile seawater. In the laboratory, clean apical fragments of the *Bryopsis* specimens were isolated and cultured in sterile 1 x modified Provasoli enriched seawater [15] at 23°C under 12h:12h (Light:Dark) conditions with a photon flux rate of 25–30 µE m^−2^s^−1^. This isolation procedure was repeated for several months until the *Bryopsis* cultures were free of eukaryotic contamination. Thus, the *Bryopsis* isolates were kept in culture for eight months prior to molecular analyses in October 2009. After isolation, all five unialgal *Bryopsis* cultures were maintained in the laboratory under the culture conditions described above.

### Fluorescence in situ hybridization

Unialgal *Bryopsis* thalli were fixed in 4% paraformaldehyde and 0.25% glutaraldehyde in 50 mM PIPES (piperazine-N,N′-bis(2-ethanesulfonic acid)) buffer, pH 7.2 for 2 hours. After dehydration through a graded ethanol series from 30% to 80%, ethanol was subsequently replaced by LR white embedding medium (London Resin, UK). Samples were loaded in gelatine capsules and allowed to polymerize at 37°C for 3 days. Semithin sections were cut using glass knives on a Microm HM360 microtome (Microm International GmbH, Germany) and collected on Vectabond-coated (Vector Laboratories, USA) slides. *In situ* hybridization was performed as described by Daims *et al.* [16] with 200 µL formamide per mL hybridization buffer, an incubation of 90 min at 46°C, and the universal bacterial Cy3-labelled EUB338 probe mix [17]. Algal DNA and cell wall counterstaining was performed by adding a mix of 4,6-diamidino-2-phenylindole (DAPI) and calcofluor to the sections for 7 min in the dark at room temperature. Sections were mounted in AF-1 antifadent (Citifluor, UK) and viewed with an Olympus BX51 epifluorescence microscope fitted with a DAPI/FITC/TRITC triple band filter. The *Bryopsis* specimens were not surface-sterilized prior to hybridization due to potential morphological losses.

### Surface sterilization, DNA extraction and PCR

To identify the endophytic bacterial diversity, approximately 2 grams (ww) of each unialgal *Bryopsis* sample was surface-sterilized as previously described [14] prior to a total DNA extraction using a CTAB protocol modified from Doyle and Doyle [18]. These extracts, containing both algal and bacterial DNA, were subjected to *rbc*L and 16S rRNA gene PCR amplifications following protocols outlined in Hanyuda *et al*. [19] and Lane [20] with, respectively, primer pairs 7F/R1391 and 27F/1492R. All obtained PCR amplicons were purified using a Nucleofast 96 PCR clean up membrane system (Machery-Nagel, Germany) according to the manufacturer's instructions.

### Cloning and DGGE

To determine the bacterial diversity, purified 16S rRNA gene amplicons from the algal extracts were cloned using the pGEM®-T Vector System (Promega Benelux, The Netherlands). For each *Bryopsis* sample a clone library of 150 clones was prepared, the diversity of which was examined via short fragment sequencing (see below). For dereplication, clones' short sequences were grouped into the same operational taxonomic unit (OTU) when having ≥97% similarity. From each OTU, representative clones were selected for full length (±1450 bp) 16S rRNA gene sequencing (see below). Clone libraries' coverage was verified by DGGE analysis of each *Bryopsis* DNA extract and its representative clones. A V3 PCR with primers F357-GC/R518 and subsequent DGGE analysis were carried out as described previously [14], with a denaturing gradient of 45–65%. DGGE banding patterns were normalized using the BioNumerics 5.1 software (Applied Maths, Belgium). DGGE bands from the algal extracts which showed no correspondence with OTU band positions were excised from the polyacrylamide gel following Van Hoorde *et al.* [21] and sequenced (±150 bp) as described below.

### Sequencing

*Rbc*L genes, DGGE bands as well as short and full length 16S rRNA genes were sequenced on an ABI PRISM 3130xl Genetic Analyzer (Applied Biosystems, USA) by means of the BigDye® xTerminator™ v.3.1 Cycle Sequencing and Purification Kit (Applied Biosystems, USA) according the protocol of the supplier. Primers used were, respectively, 7F/R1391 [19], F357/R518 [21], BKL1 [22] and T7/SP6 (Promega Benelux, The Netherlands). Sequences obtained were assembled in BioNumerics, compared with nucleotide databases via BLAST and chimera-checked using Bellerophon [23]. The bacterial 16S rRNA gene and *Bryopsis* chloroplast 16S rRNA gene and *rbc*L sequences were submitted to GenBank under accession numbers JF521593-JF521615 (see also Table 1).

**Table 1:** Taxonomic affiliation of the clones representing the bacterial OTUs, sorted per *Bryopsis* sample.

Host		16S rRNA gene sequence analysis of bacterial clones						
*Bryopsis* sample	Chloroplast 16S rRNA gene^1^ and *rbc*L accession no.	OTU no.^2^	OTU representative clone name	Accession no.	OTU library %/sample^3^	Higher taxonomic ranks	Three closest NCBI matches	Accession no. (Query coverage/Maximum identity)
MX19	JF521612JF521594	OTU-3	MX19.8	JF521598	0.8%	Bacteroidetes; unclassified Bacteroidetes	Uncultured bacterium clone Dstr_N15	GU118164 (99/94)
							Uncultured bacterium clone SGUS845	FJ202831 (100/92)
							Endosymbiont of *Acanthamoeba* sp. KA/E21	EF140637 (100/91)
		OTU-2	MX19.9	JF521606	14.2%	Tenericutes, Mollicutes, Mycoplasmatales, Mycoplasmataceae	Uncultured bacterium clone GB96	GU070687 (100/97)
							Uncultured bacterium clone frc89	HQ393440 (100/93)
							Uncultured bacterium isolate SRODG064	FM995178 (100/90)
		OTU-4	MX19.12	JF521607	3%	Proteobacteria; Alphaproteobacteria; Rhizobiales; Phyllobacteriaceae	Uncultured Rhizobiales bacterium clone PRTBB8661	HM799061 (99/99)
							Uncultured Rhizobiaceae bacterium clone TDNP_Wbc97_42_3_189	FJ517108 (100/97)
							Uncultured alpha proteobacterium clone D2F10	EU753666 (100/97)
		OTU-1	MX19.14	JF521603	2.3%	Bacteroidetes; Flavobacteria; Flavobacteriales	Uncultured bacterium clone SHFH601	FJ203530 (99/96)
							Uncultured Bacteroidetes bacterium clone CN77	AM259925 (100/94)
							Uncultured bacterium clone SINP825	HM127741 (99/89)
MX90	JF521615JF521597	OTU-1	MX90.40	JF521602	6.5%	Bacteroidetes; Flavobacteria; Flavobacteriales	Uncultured bacterium clone SHFH601	FJ203530 (99/96)
							Uncultured Bacteroidetes bacterium clone CN77	AM259925 (100/94)
							Uncultured bacterium clone SINP825	HM127741 (99/88)
MX164	JF521611JF521593	OTU-5	MX164.9	JF521609	63.6%	Proteobacteria; Gammaproteobacteria; Xanthomonadales; Xanthomonadaceae	Gamma proteobacterium strain OS-28	EF612351 (100/94)
							Uncultured *Luteibacter* sp. clone SMa210	AM930508 (100/94)
							“*Luteibacter jiangsuensis*” JW-64-1	FJ848571 (100/93)
		OTU-1	MX164.14	JF521600	7.1%	Bacteroidetes; Flavobacteria; Flavobacteriales	Uncultured bacterium clone SHFH601	FJ203530 (99/96)
							Uncultured Bacteroidetes bacterium clone CN77	AM259925 (100/94)
							Uncultured bacterium clone SINP825	HM127741 (99/89)
		OTU-6	MX164.20	JF521610	3.6%	Proteobacteria; Epsilonproteobacteria; Campylobacterales; Campylobacteraceae	*Arcobacter marinus* type strain CL-S1T	EU512920 (96/93)
							“*Arcobacter molluscorum*” type strain CECT7696T	FR675874 (94/94)
							Uncultured *Arcobacter* sp. clone bo13C09	AY862492 (96/93)
		OTU-4	MX164.59	JF521608	5%	Proteobacteria; Alphaproteobacteria; Rhizobiales; Phyllobacteriaceae	Phylobacteriaceae bacterium strain DG943	AY258089 (97/99)
							Uncultured bacterium clone Apal_F11	GU118131 (99/98)
							Uncultured bacterium clone MSB-2G6	EF125460 (100/97)
MX263	JF521613JF521595	OTU-2	MX263.1	JF521605	22.6%	Tenericutes, Mollicutes, Mycoplasmatales, Mycoplasmataceae	Uncultured bacterium clone GB96	GU070687 (100/97)
							Uncultured bacterium clone frc89	HQ393440 (100/93)
							Uncultured bacterium isolate SRODG064	FM995178 (100/90)
		OTU-1	MX263.61	JF521604	4%	Bacteroidetes; Flavobacteria; Flavobacteriales	Uncultured bacterium clone SHFH601	FJ203530 (99/96)
							Uncultured Bacteroidetes bacterium clone CN77	AM259925 (100/94)
							Uncultured bacterium clone SINP825	HM127741 (99/89)
		OTU-3	MX263.73	JF521599	1.4%	Bacteroidetes; unclassified Bacteroidetes	Uncultured bacterium clone Dstr_N15	GU118164 (99/94)
							Uncultured bacterium clone SGUS845	FJ202831 (100/92)
							Endosymbiont of *Acanthamoeba* sp. KA/E21	EF140637 (100/91)
MX344	JF521614JF521596	OTU-1	MX344.2	JF521601	2.2%	Bacteroidetes; Flavobacteria; Flavobacteriales	Uncultured bacterium clone SHFH601	FJ203530 (99/96)
							Uncultured Bacteroidetes bacterium clone CN77	AM259925 (100/94)
							Uncultured bacterium clone SINP825	HM127741 (99/89)

1 Chloroplast 16S rRNA gene sequences were derived from clones MX19.1, MX90.9, MX164.1, MX263.48 and MX344.10 with an OTU library percentage of, respectively, 79.7, 93.5, 20.7, 68 and 97.8 percent per sample.

2 All bacterial OTUs containing clones derived from different *Bryopsis* strains had minimal intra-OTU sequence similarities of ≥97% ranging from exactly 97% in OTU-4, over 99.3% and 99.7% in, respectively, OTU-2 and OTU-1, to no less than 99.9% pairwise similarity in OTU-3.

3 Especially noteworthy is the abundance of OTU-5 in the MX164 sample's clone library. While the bacterial OTUs 1, 3, 4 and 6 have a low occurrence of 0.8–7.1% and OTU-2 a considerable presence of 14.2–22.6% in their respective clone libraries, OTU-5 amounts to a substantial percentage (63.6%) of the MX164 sample's clones. In addition, only *Bryopsis* sample MX263 comprised chimeric Flavobacteriaceae-*Bryopsis* chloroplast 16S rRNA gene sequences which made up 4% of the sample's clone library.

### Phylogenetic analyses

Two sets of alignments, made using MUSCLE [24], were considered for phylogenetic analyses. The first one, consisting of a concatenated chloroplast 16S rRNA gene and *rbc*L dataset, was used for the creation of a *Bryopsis* phylogram. A second set of alignments was assembled to assess 16S rRNA gene phylogenetic relationships between the *Bryopsis*-associated bacterial endophytes and known bacterial species, including BLAST hits and algae-associated bacteria described in the literature. The most suitable model for phylogenetic analysis was selected using the AIC criterion in jModelTest [25]. Subsequently, the *Bryopsis* host and bacterial datasets were analyzed by means of the maximum likelihood (ML) algorithm in PhyML v3.0 [26] under a HKY + G4 model via the University of Oslo Bioportal website (http://www.bioportal.uio.no//). Reliability of ML trees was evaluated based on 100 bootstrap replicates. Output ML trees were subsequently visualized in Mega 4.0 [27] and edited with Adobe® Illustrator® CS5.

## Results

### Fluorescence in situ hybridization

**Figure 2:**
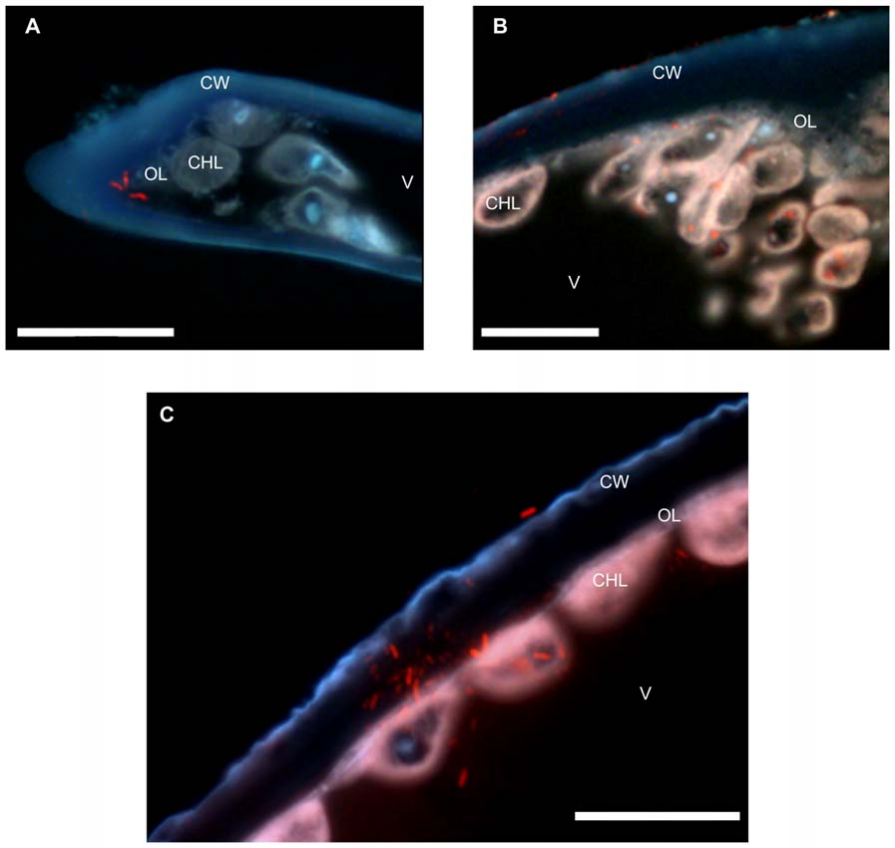
Epifluorescence microscopy images of *Bryopsis* sections hybridized with the universal bacterial Cy3-EUB338 probe mix (red). DAPI (light blue) and calcofluor (dark blue) were used as counter stains to visualize algal DNA in nuclei and chloroplasts and the algal cell wall, respectively. Metabolically active bacteria (red) are present throughout the *Bryopsis* cytoplasm: in the outer layer (OL) next to the cell wall (CW) which contains most of the organelles like mitochondria, endoplasmic reticulum, and nuclei (A–C), and in the inner chloroplast layer (CHL) immediately adjacent to the vacuole (V) (B–C). Since the *Bryopsis* thalli were not surface sterilized before fixation, the red probe also hybridized with epiphytic bacteria on the calcofluor stained cell wall (B–C). The scale bar on all images is 20 µm.

To confirm the observation of endogenous bacteria in *Bryopsis* made by Burr and West [5], *Bryopsis* sections were hybridized with the universal bacterial EUB338 probe mix labelled with Cy3. Figures 2A–C depict clear binding of the red fluorescent probe mix to bacterial rRNA present throughout the cytoplasm; both in the outer layer next to the cell wall, which contains most of the organelles except the chloroplasts (Figures 2A–C), as well as in the inner chloroplast layer immediately adjacent to the vacuole (Figures 2B–C). These hybridization results demonstrate the presence of metabolically active bacteria within the *Bryopsis* cytoplasm. Since the *Bryopsis* thalli were not surface sterilized before fixation, the EUB338 probe mix also hybridized with epiphytic bacterial rRNA on the cell wall (Figures 2B–C).

### Bacterial diversity within Bryopsis algae: Cloning

Five clone libraries were created using the amplified 16S rRNA gene fragments from samples MX19, MX90, MX164, MX263 and MX344. After clone dereplication, 16S rRNA gene sequences from all five clone libraries covered no more than seven unique OTUs. By far the most common OTU, representing 72% of the total clones screened, showed ≥96% sequence similarity with the *B. hypnoides* chloroplast 16S ribosomal RNA gene (AY221722). The six remaining OTUs, on the other hand, contained bacterial sequences belonging to the phyla Bacteroidetes, Proteobacteria or Tenericutes (Table 1). OTU-1 was detected in all five *Bryopsis* cultures and had 96% sequence similarity with an uncultured Flavobacteriales bacterium (FJ203530) associated with the coral *Montastraea faveolata*. OTU-2 and 3 were only present in the *B*. *hypnoides* samples. OTU-2 is related to uncultured Mycoplasmataceae bacteria isolated from the intestine of the small abalone *Haliotis diversicolor* (GU070687, HQ393440). OTU-3 is allied to unclassified Bacteroidetes bacteria associated with corals (GU118164, FJ202831) or *Acanthamoeba* species (EF140637). OTU-4 sequences were detected in cultures MX19 and MX164, and showed high similarity (≥97%) with Phyllobacteriaceae bacteria isolated from seawater (HM799061, FJ517108), dinoflagellates (AY258089), stromatolites (EU75366) or corals (GU118131). OTU-5 and 6 were only present in *B*. *pennata* var. *leprieurii* sample MX164 and are distantly related (93–94%) to, respectively, *Luteibacter* sp. (Xanthomonadaceae) present in soil (EF612351, AM930508, FJ848571) and *Arcobacter* strains (Campylobacteraceae) recovered from mussels (FR675874) and seawater surrounding seaweeds and starfish (EU512920).

### Bacterial diversity within Bryopsis algae: DGGE

**Figure 3:**
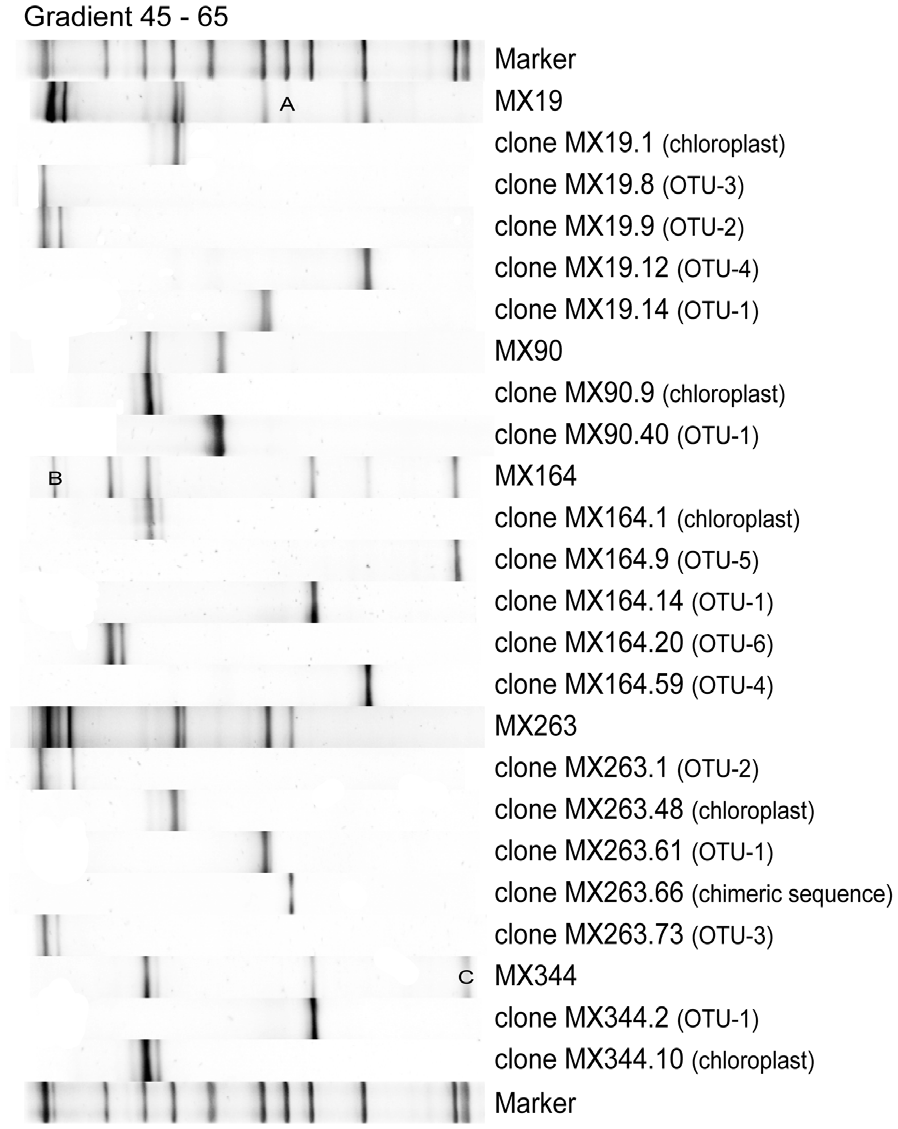
Normalized DGGE profiles of MX DNA extracts and their representative OTUs. DGGE bands marked with letters A, B and C, which did not match any of the individual OTU bands, were excised from the polyacrylamide gel and sequenced. The first and last lanes contain a known molecular marker used for normalization.

Coverage of the clone libraries was verified by comparing DGGE community profiles of the different *Bryopsis* DNA extracts with the banding pattern of clones from their respective OTUs, including representative clones with 16S rRNA gene chloroplast and chimeric sequences. As shown in Figure 3 the OTUs DGGE bands overlap well with the individual bands of the MX extracts' DGGE profiles, indicating adequate clone library coverage. MX samples 19, 164 and 344, however, all showed one band in their DGGE profile not represented by an OTU band. Consequently, these three DGGE bands (A, B and C, respectively) were excised and sequenced. The sequence of DGGE band A showed 100% similarity with the chimeric sequences detected in MX sample 263, not unexpected given its corresponding band position with clone MX263.66. DGGE band B was identified as forming part of the OTU-2 cluster with 100% sequence similarity with clone MX19.9, whereas DGGE band C showed no correspondence with any bacterial OTU detected. Hence, the latter DGGE band was assigned to a new OTU, i.e. OTU-7. BLAST searches revealed that this OTU-7 is closely related to *Labrenzia* species isolated from the green seaweed *Ulva rigida* (FN811315), crustose coralline red algae (HM178529) and the dinoflagellate *Karlodinium micrum* (HM584720).

**Figure 4:**
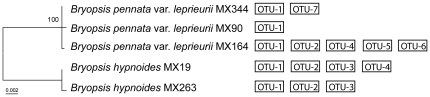
Endophytic diversity results (right) plotted against the *Bryopsis* host phylogeny (left). The OTU diversity (1–7) displayed on the right summarizes the diversity results from the clone libraries and DGGE analyses. The concatenated chloroplast 16S rRNA gene - *rbc*L maximum likelihood tree on the left classifies the *Bryopsis* MX samples in two distinct species clades with 100% bootstrap support. The scale bar indicates 0.002 nucleotide changes per nucleotide position.

Figure 4 depicts the endophytic diversity results from the clone libraries and DGGE analyses plotted on a phylogram representing the relations between the five *Bryopsis* samples. From Figure 4 we can deduce that Flavobacteriaceae (OTU-1), *Mycoplasma* (OTU-2), Bacteroidetes (OTU-3) and Phyllobacteriaceae (OTU-4) species were present in more than one *Bryopsis* sample examined. Even though the endobiotic community members were to a certain extent similar, the total diversity of the endophytic community was unique to each *Bryopsis* sample. None of the *Bryopsis* samples harbored the same number or range of bacterial endophytes.

### Bacterial diversity within Bryopsis algae: Phylogenetic analysis

**Figure 5:**
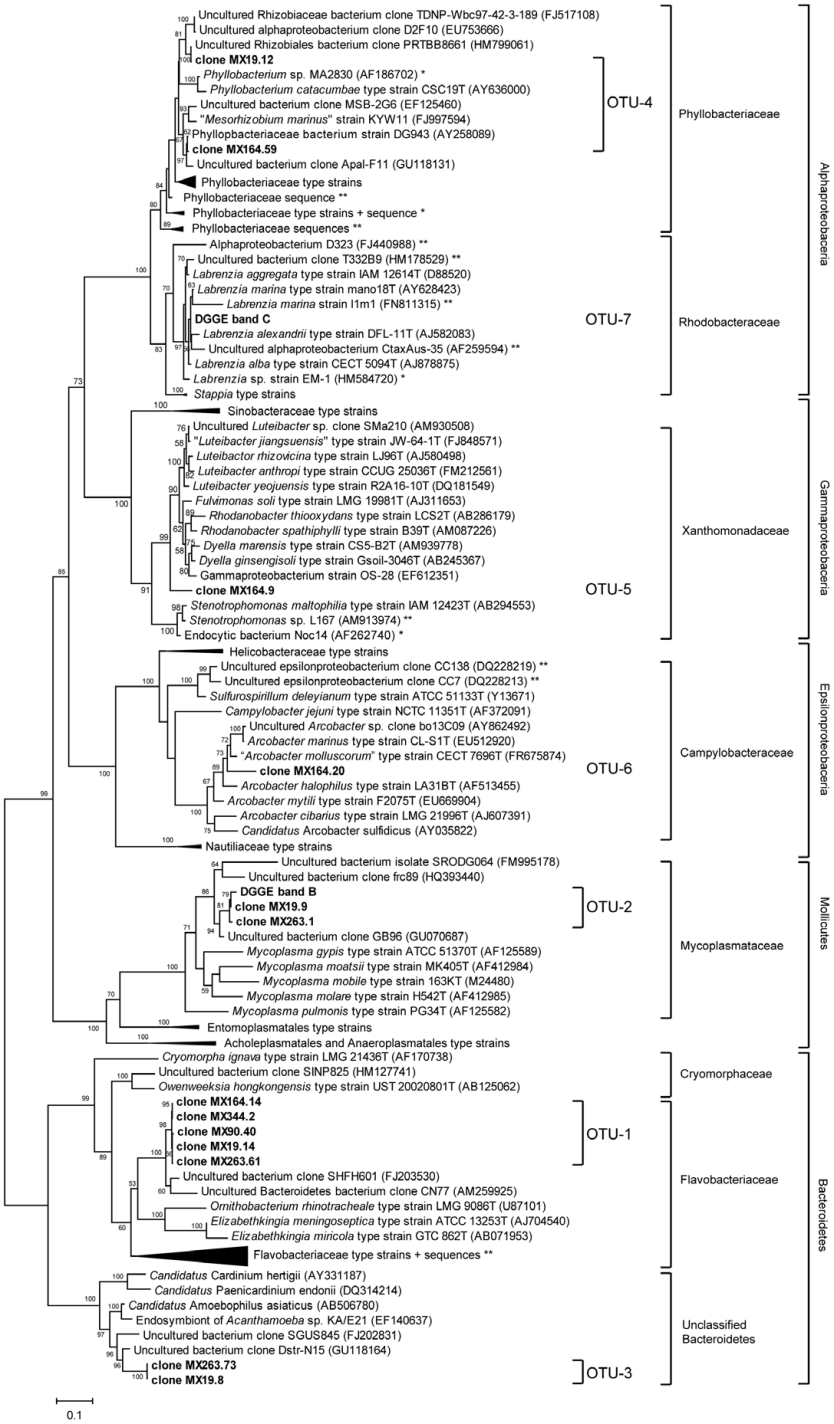
A wide-range maximum likelihood tree showing the phylogenetic positions of endophytic clones and DGGE bands. Phylogenies were inferred from 16S rRNA gene sequences determined in this study (in bold), BLAST hits (see Table 1), Bacteroidetes, Proteobacteria and Mollicutes type strains, and algae-associated bacteria described in the literature (see Table S1). The tree was generated in PhyML according the HKY + G4 algorithmic model. Bootstrap values above 50% are indicated at the branch nodes and the scale bar shows 10 nucleotide substitutions per 100 nucleotides. Asterisks denote sequences previously isolated from micro * - and macroalgae**. The same phylogenetic tree without compressed branches is depicted in Figure S1.

A wide-range phylogenetic tree (Figure 5, tree without compressed branches see Figure S1) was created, which includes bacterial OTUs determined in this study (clones and DGGE bands), significant BLAST hits (Table 1), type strains from the Bacteroidetes, Proteobacteria and Tenericutes division, and algae-associated bacteria described in the literature (Table S1). As could be predicted from the BLAST maximum identity scores (Table 1), none of the endobiotic bacterial sequences clustered tightly with cultivated bacterial type strains. Consequently, all endophytic bacterial OTUs derived from *Bryopsis* represent new species or genera which in some cases match previously sequenced unclassified bacteria. These OTU sequences, however, all showed at least 93% sequence similarity with their best BLAST hit which generally resulted in phylogenetic placements with good bootstrap support. Accordingly, all OTU-1 sequences formed a distinct and well-supported (98%) clade within the Flavobacteriaceae family and most likely represent a new genus given their low sequence similarities (87% at most) with Flavobacteriaceae type strains. The similarity among the five OTU-1 sequences, however, was 99.7%, suggesting all sequences belong to the same new Flavobacteriaceae genus even though they were derived from different *Bryopsis* samples collected several hundred kilometres apart. Likewise, the Bacteroidetes OTU-3 clones were virtually identical displaying 99.9% pairwise similarity. These OTU-3 clones, found in *B. hypnoides* samples MX19 and MX263, belong to a single clade (100% bootstrap support) of unclassified Bacteroidetes, but are distantly related to other unclassified Bacteroidetes symbionts. The OTU-2 clade, consisting of clones MX19.9 and MX263.1 and DGGE band B, fell into the genus *Mycoplasma* with 100% bootstrap support although these clones showed low levels of similarity (≤90%) with *Mycoplasma* type strains. All OTU-2 sequences presumably belong to one and the same new *Mycoplasma* species (99.7 intra-OTU sequence similarity). The majority of the endophytic bacterial OTUs, however, were affiliated with the Proteobacteria phylum and belonged to the Alpha-, Gamma- and Epsilonproteobacteria. Particularly, OTU-5 and 6, both consisting of clones exclusively obtained from *B. pennata* var. *leprieurii* sample MX164, most probably represent a new genus of Xanthomonadaceae and a new *Arcobacter* species, respectively. OTU-4 and 7 are robustly affiliated (100% bootstrap support) with the Alphaproteobacteria class and belong to the Rhizobiales and Rhodobacterales, respectively. Despite the high sequence similarity of OTU-7 with algal-associated *Labrenzia* species, relatedness of DGGE band C with the *Labrenzia alexandrii* type strain (AJ582083) and an uncultured *Labrenzia* bacterium isolated from *Caulerpa taxifolia* (AF259594) lacks bootstrap support. The shortness of the DGGE band C sequence (±150 bp) and, consequently, the poor resolution within this clade, made it difficult to conclude whether OTU-7 represents a new *Labrenzia* species. Finally, OTU-4 is the only OTU containing clones derived from different *Bryopsis* samples in which the representative clones, i.e. clone MX19.12 and MX164.59, did not cluster together. This is in agreement with the 97% intra-OTU sequence similarity. Hence, both clones belong to the Phyllobacteriaceae clade with good bootstrap support (80%), but most likely represent two different new species or genera because of their low sequence similarities (96% at most) with Phyllobacteriaceae type strains.

## Discussion

Forty years after Burr and West [5] observed endogenous ‘bacteria-like particles’ in *Bryopsis hypnoides*, this is the first study to verify the presence of metabolically active endophytic bacteria inside the *Bryopsis* cytoplasm by means of the FISH technique. Mainly due to the intense background autofluorescence of algal cells, reports of successful FISH applications on macroalgae are limited to analyses of macroalgal surface-associated bacteria [28] and algal gall endosymbionts [4]. The use in this study of semithin algal sections and a triple band filter, however, made it possible to discriminate bacterial FISH signals from autofluorescence of algal pigments using standard FISH protocols in combination with epifluorescence microscopy. Even though *Bryopsis* samples were not surface-sterilized prior to hybridization to avoid potential morphological losses, the solid embedding at the start of the FISH protocol proved successful in immobilizing the epiphytes on the *Bryopsis* surface (data not shown). This prevented the detachment and potential spread of surface bacteria during sectioning. Consequently, our FISH results strongly suggest the presence of bacteria within *Bryopsis* cells.

In this study, the first insights are provided into the identity and phylogenetic diversity of endobiotic bacterial communities within *Bryopsis*. Despite the limited number of samples studied, our results indicate that *Bryopsis* harbors endophytic bacterial communities which are not very complex (i.e. only 7 bacterial OTUs detected), but taxonomically diverse including *Arcobacter*, Bacteroidetes, Flavobacteriaceae, *Mycoplasma*, *Labrenzia*, Phyllobacteriaceae and Xanthomonadaceae species. Although the composition of the total endophytic community seems unique to each *Bryopsis* culture, Bacteroidetes, Flavobacteriaceae, *Mycoplasma* and Phyllobacteriaceae species were detected in two or more *Bryopsis* samples. In particular OTU-1 Flavobacteriaceae species are present in all five *Bryopsis* cultures, which were collected from diverse sites along the Mexican west coast. Delbridge and colleagues [10] made similar observations when comparing the endosymbiotic communities within four different *Caulerpa* species. While the endosymbiotic communities seemed unique to each *Caulerpa* individual, all community members were photosynthetic Alphaproteobacteria.

Also within *Bryopsis*, Alphaproteobacteria appear well represented. This is not unexpected, since Alphaproteobacteria are frequently associated with macroalgae [1], [29], [30], an alliance which may be linked to dimethylsulfoniopropionate (DMSP) exchange [31]. Particularly OTU-7, belonging to the marine phototrophic and CO-oxidizing *Labrenzia* genus [32], [33], is closely related to an uncultured bacterium reported by Meusnier *et al.* [29] in their study on the total bacterial community associated with *Caulerpa taxifolia*. Although *Labrenzia* species have not been reported as endophytes, the presence of Rhizobiales-specific proteins in *L. aggregata* [34] may hint at potential endosymbiotic features. The Rhizobiales order contains various well-known nitrogen fixing plant symbionts, mainly in terrestrial habitats but also in marine environments [35]. Moreover, Rhizobiales bacteria are common epiphytes on green [31], [36], brown [37], [38] and red [36] macroalgae; and a *Rhodopseudomonas* species with the potential to fix nitrogen was isolated from the inside of *C. taxifolia* [9]. Also within *Bryopsis*, Rhizobiales species seem to be well established as clones MX19.12 and MX164.59 (OTU-4) likely represent two different new Phyllobacteriaceae species or genera clustering together with, respectively, a free-living marine Phyllobacteriaceae bacterium [39] and a dinoflagellate-associated anoxygenic photosynthetic bacterial strain [40]. In addition, we amplified a Phyllobacteriaceae nitrogenase-like light-independent protochlorophyllide reductase gene (submitted to GenBank under accession number JN048464) from *Bryopsis* sample MX164 by the *nif*H protocol described by De Meyer *et al.* [41], supporting the above suggested relatedness of OTU-4 to photosynthetic bacteria.

Besides the presence of Alphaproteobacteria in three of the five *Bryopsis* cultures studied, endophytes from the Gamma- and Epsilonproteobacteria order seem restricted to a single *Bryopsis* sample. The latter endophytes (OTU-6) most likely belong to a new *Arcobacter* species within the Campylobacteraceae family. *Arcobacter* species are mainly known as potential human and animal pathogens, but have also been isolated from diverse marine environments including seawater surrounding seaweeds [42], [43]. Despite their ecologically significant functions like nitrogen fixation, denitrification, sulfide oxidation and manganese reduction [42], [44], they are not frequently reported as endobionts [45], [46]. On the other hand, members of the Xanthomonadaceae family to which OTU-5 belongs, are well-known plant endophytes [47] and have previously been isolated from marine algae [38], [48]. Since many Xanthomonadaceae species cause plant diseases, the high number of Xanthomonadaceae endophytes within *Bryopsis* MX164 could be a sign of infection. The alga, however, showed no visible disease symptoms (e.g. bleaching), indicating a neutral or beneficial relationship.

In the Bacteroidetes group, we found two distinct clusters (i.e. OTU-1 and OTU-3) of endophytic bacteria, one within the Flavobacteriaceae family and one belonging to unclassified Bacteroidetes. The Flavobacteriaceae endophytes (OTU-1) show an especially strong association with *Bryopsis* as evidenced by their occurrence in all five samples. The phylum Bacteroidetes, and in particular the family Flavobacteriaceae, forms one of the major components of marine bacterioplankton and mediates a substantial proportion of the carbon flow and nutrient turnover in the sea during and following algal blooms [49]. Moreover, many novel Bacteroidetes members, some of which were characterized as morphogenesis inducers [50], have been isolated from the surfaces of marine macroalgae [1]. Whereas Bacteroidetes bacteria are obviously common epiphytes on macroalgae, Meusnier and co-workers [29] suggested the existence of an endophytic Cytophaga-Flavobacteria-Bacteroidetes (CFB) bacterium within *Caulerpa taxifolia*. In addition, Bacteroidetes bacteria are well-known endosymbionts of amoebae, plant-parasitic nematodes and insects [51]–[53]. Phylogenetic analysis, however, revealed that the Bacteroidetes endophytes of *Bryopsis* are more closely related to bacteria tightly associated with corals and sponges [54]–[56] than to CFB sequences isolated from green [29], [50], brown [38] and red [57], [58] macroalgae.

Finally, three *Bryopsis* samples (i.e. MX19, 164 and 263) contained *Mycoplasma* sequences (OTU-2). Mycoplasmas are well-known human and animal parasites, but are also common members of the intestinal bacterial flora of fishes and abalones where they may provide nutrients to their hosts [46], [59], [60]. Moreover, the close affiliation of *Mycoplasma* sequences isolated from *Bryopsis* and abalone species is perhaps not at all surprising as the latter generally feeds on a broad selection of algae [61]. Also Huang and colleagues [59] postulated that the presence of *Mycoplasma* species in the intestinal microflora of the abalone *Haliotis diversicolor* could be algal-food related. Additionally, this bacterial link between *Bryopsis* and abalone species might be extrapolated to other marine gastropod mollusks, supporting the hypothesis of Rao *et al.* [13] that the production of therapeutic kahalalides by the sea slug *Elysia rufescens* as well as by its *Bryopsis* food could actually be performed through an associated microorganism. Indeed, it has been shown that several metabolites initially assigned to eukaryotes are in fact of microbial origin [1].

In summary, molecular analysis revealed, for the first time, that *Bryopsis* harbors relatively restricted but taxonomically diverse communities of endophytic bacteria. The presence of Phyllobacteriaceae, Bacteroidetes, *Mycoplasma*, and in particular Flavobacteriaceae endophytes in several *Bryopsis* samples collected hundreds of kilometres apart indicates a close association between these endophytes and *Bryopsis* plants. Even though these endophytic bacterial communities within *Bryopsis* cultures might not fully represent those that are present within the alga in its natural environment, the bacteria identified in this study are at least part of the natural *Bryopsis* endobiotic flora. Future investigations of *Bryopsis* algae in natural environments, however, are necessary to complete the *Bryopsis*-bacterial endobiosis picture.

## Supporting Information

Table S1 **Bacterial 16S rRNA gene sequences isolated from algae (excluding BLAST hits) included in the phylogenetic analysis.**
(DOCX)

Figure S1 **A wide-range maximum likelihood tree showing the phylogenetic positions of endophytic clones and DGGE bands.** Phylogenies were inferred from 16S rRNA gene sequences determined in this study (in bold), BLAST hits (see Table 1), Bacteroidetes, Proteobacteria and Mollicutes type strains, and algae-associated bacteria described in the literature (see Supplementary Table S2). The tree was generated in PhyML according the HKY + G4 algorithmic model. Bootstrap values above 50% are indicated at the branch nodes and the scale bar shows 10 nucleotide substitutions per 100 nucleotides. Asterisks denote sequences previously isolated from micro * - and macroalgae**.
(TIF)
