# Supplementary material for: Who Is in There? Exploration of Endophytic Bacteria within the Siphonous Green Seaweed Bryopsis (Bryopsidales, Chlorophyta)
Source: PLoS One. 2011 Oct 18;6(10):e26458. doi: 10.1371/journal.pone.0026458 (PMC3196581; doi:10.1371/journal.pone.0026458)
Supplement: Table S1 — Bacterial 16S rRNA gene sequences isolated from algae (excluding BLAST hits) included in the phylogenetic analysis. (DOCX) [file pone.0026458.s001.docx]

| **Table S1: Bacterial 16S rRNA gene sequences isolated from algae (excluding BLAST hits) included in the phylogenetic analysis** | | | | |
| --- | --- | --- | --- | --- |
| **Higher taxonomic ranks** | **Taxon label** | **Accession no.** | **Isolation source** | **Reference** |
| Bacteroides | Bacteroidetes bacterium D295 | FJ440982 | *Delisea pulchra* | [[1](#_ENREF_1)] |
|  | Bacteroidetes bacterium LD83 | AM913945 | *Saccharina latissima* | [[2](#_ENREF_2)] |
|  | Flavobacteriaceae bacterium Cv4 | EU278337 | *Ceramium virgatum* | [[3](#_ENREF_3)] |
|  | Flavobacteriaceae bacterium I-1856 | AB073589 | Green macroalga | [[4](#_ENREF_4)] |
|  | Flavobacteriaceae bacterium Pf4 | EU278339 | *Polysiphonia fucoides* | [[3](#_ENREF_3)] |
|  | Flavobacteriaceae bacterium Rc6 | EU278338 | *Rhodomela confervoides* | [[3](#_ENREF_3)] |
|  | Uncultured CFB bacterium CtaxAus-4 | AF259600 | *Caulerpa taxifolia* | [[5](#_ENREF_5)] |
|  | Uncultured CFB bacterium CtaxMed-5 | AF259614 | *Caulerpa taxifolia* | [[5](#_ENREF_5)] |
| Alphaproteobacteria | Alphaproteobacterium D323 | FJ440988 | *Delisea pulchra* | [[1](#_ENREF_1)] |
|  | Alphaproteobacterium L96 | AM913948 | *Saccharina latissima* | [[2](#_ENREF_2)] |
|  | Alphaproteobacterium RSHD3S10 | AF190214 | *Pfiesteria*-like dinoflagellate | [[6](#_ENREF_6)] |
|  | *Phyllobacterium* sp. MA2830 | AF186702 | *Pfiesteria*-like dinoflagellate | [[6](#_ENREF_6)] |
|  | Uncultured Alphaproteobacterium clone LSBS121 | AM745991 | *Saccharina latissima* | [[7](#_ENREF_7)] |
|  | Uncultured Alphaproteobacterium isolate DGGE band UA10 | DQ229324 | *Ulva australis* | [[8](#_ENREF_8)] |
|  | Uncultured bacterium clone OTU109 | GU451440 | *Gracilaria vermiculophylla* | [[9](#_ENREF_9)] |
|  | Uncultured bacterium clone OTU297 | GU451628 | *Ulva intestinalis* | [[9](#_ENREF_9)] |
|  | Uncultured Alphaproteobacterium CtaxAus-35 | AF259594 | *Caulerpa taxifolia* | [[5](#_ENREF_5)] |
| Gammaproteobacteria | Endocytic bacterium Noc14 | AF262740 | *Noctiluca scintillans* | [[10](#_ENREF_10)] |
|  | *Stenotrophomonas* sp. L167 | AM913974 | *Saccharina latissima* | [[2](#_ENREF_2)] |
| Epsilonproteobacteria | Uncultured Epsilonproteobacterium clone CC7 | DQ228213 | *Cladophora* mats | [[11](#_ENREF_11)] |
|  | Uncultured Epsilonproteobacterium clone CC38 | DQ228219 | *Cladophora* mats | [[11](#_ENREF_11)] |

1. Penesyan A, Marshall-Jones Z, Holmstrom C, Kjelleberg S, Egan S (2009) Antimicrobial activity observed among cultured marine epiphytic bacteria reflects their potential as a source of new drugs. FEMS Microbiol Ecol 69: 113-124.

2. Wiese J, Thiel V, Nagel K, Staufenberger T, Imhoff JF (2009) Diversity of antibiotic-active bacteria associated with the brown alga *Laminaria saccharina* from the Baltic Sea. Mar Biotechnol 11: 287-300.

3. Nylund GM, Cervin G, Persson F, Hermansson M, Steinberg PD, et al. (2008) Seaweed defence against bacteria: a poly-brominated 2-heptanone from the red alga *Bonnemaisonia hamifera* inhibits bacterial colonisation. Mar Ecol Prog Ser 369: 39-50.

4. Matsuo Y, Suzuki M, Kasai H, Shizuri Y, Harayama S (2003) Isolation and phylogenetic characterization of bacteria capable of inducing differentiation in the green alga *Monostroma oxyspermum*. Environ Microbiol 5: 25-35.

5. Meusnier I, Olsen JL, Stam WT, Destombe C, Valero M (2001) Phylogenetic analyses of *Caulerpa taxifolia* (Chlorophyta) and of its associated bacterial microflora provide clues to the origin of the Mediterranean introduction. Mol Ecol 10: 931-946.

6. Alavi M, Miller T, Erlandson K, Schneider R, Belas R (2001) Bacterial community associated with *Pfiesteria*-like dinoflagellate cultures. Environ Microbiol 3: 380-396.

7. Staufenberger T, Thiel V, Wiese J, Imhoff JF (2008) Phylogenetic analysis of bacteria associated with *Laminaria saccharina*. FEMS Microbiol Ecol 64: 65-77.

8. Tujula NA, Crocetti GR, Burke C, Thomas T, Holmstrom C, et al. (2010) Variability and abundance of the epiphytic bacterial community associated with a green marine Ulvacean alga. ISME J 4: 301-311.

9. Lachnit T, Meske D, Wahl M, Harder T, Schmitz R (2011) Epibacterial community patterns on marine macroalgae are host-specific but temporally variable. Environ Microbiol 13: 655-665.

10. Seibold A, Wichels A, Schütt C (2001) Diversity of endocytic bacteria in the dinoflagellate *Noctiluca scintillans*. Aquat Microb Ecol 25: 229-235.

11. Olapade OA, Depas MM, Jensen ET, McLellan SL (2006) Microbial communities and fecal indicator bacteria associated with *Cladophora* mats on beach sites along Lake Michigan shores. Appl Environ Microbiol 72: 1932-1938.
