# Supplementary figures and images for: Who Is in There? Exploration of Endophytic Bacteria within the Siphonous Green Seaweed Bryopsis (Bryopsidales, Chlorophyta)
Source: PLoS One. 2011 Oct 18;6(10):e26458. doi: 10.1371/journal.pone.0026458 (PMC3196581; doi:10.1371/journal.pone.0026458)

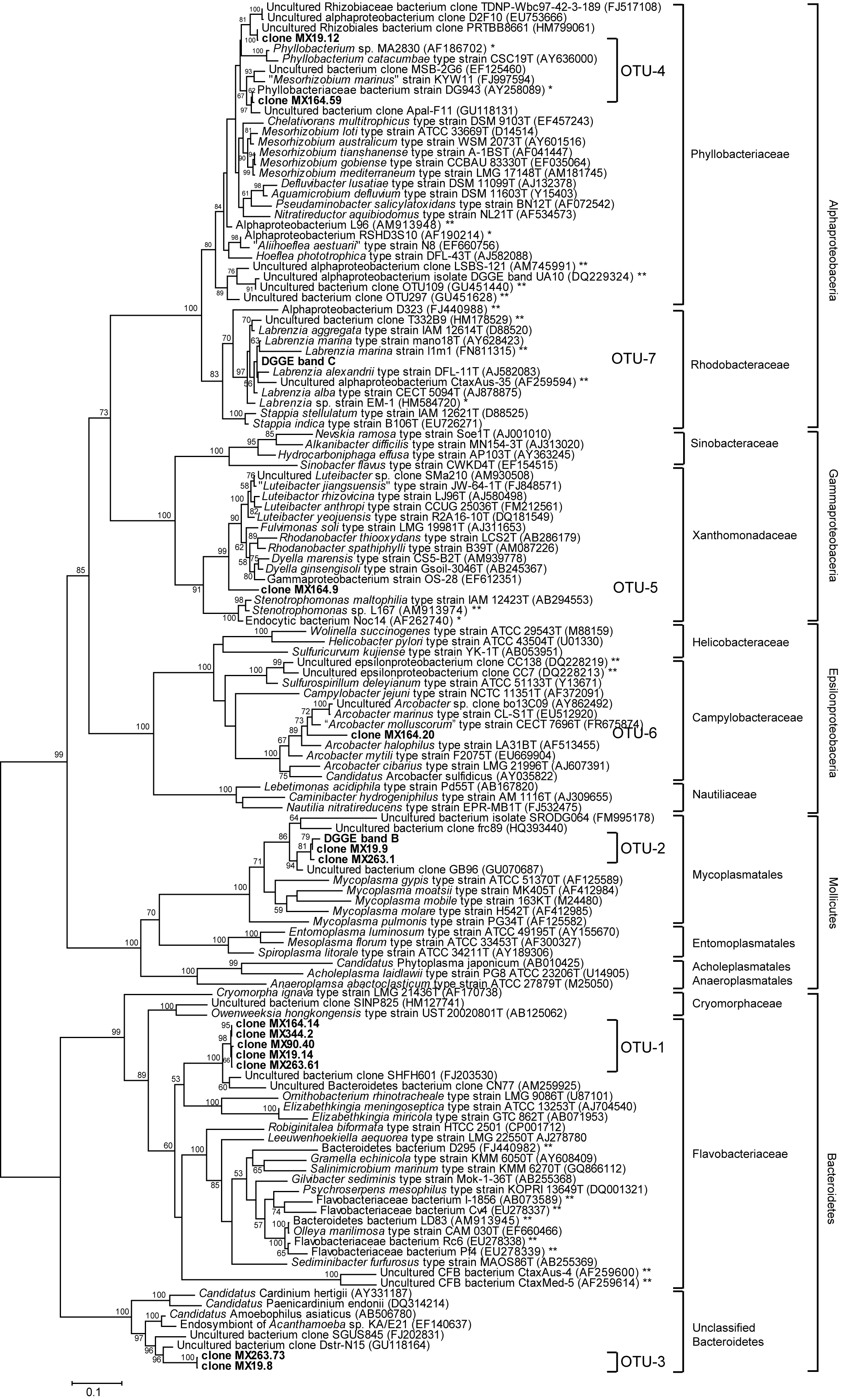

Supplement: Figure S1 — A wide-range maximum likelihood tree showing the phylogenetic positions of endophytic clones and DGGE bands. Phylogenies were inferred from 16S rRNA gene sequences determined in this study (in bold), BLAST hits (see Table 1), Bacteroidetes, Proteobacteria and Mollicutes type strains, and algae-associated bacteria described in the literature (see Supplementary Table S2). The tree was generated in PhyML according the HKY + G4 algorithmic model. Bootstrap values above 50% are indicated at the branch nodes and the scale bar shows 10 nucleotide substitutions per 100 nucleotides. Asterisks denote sequences previously isolated from micro * - and macroalgae**. (TIF) [file pone.0026458.s002.tif]
